# A Study on Energy Consumption in AI-Driven Medical Image Segmentation

**DOI:** 10.3390/jimaging11060174

**Published:** 2025-05-26

**Authors:** R. Prajwal, S. J. Pawan, Shahin Nazarian, Nicholas Heller, Christopher J. Weight, Vinay Duddalwar, C.-C. Jay Kuo

**Affiliations:** 1 Radiomics Lab, University of Southern California, Los Angeles, CA 90033, USA; prajwalr0411@gmail.com (R.P.); vinay.duddalwar@med.usc.edu (V.D.); 2 Ming Hsieh Department of Electrical and Computer Engineering, University of Southern California, Los Angeles, CA 90089, USA; shahin.nazarian@usc.edu (S.N.); jckuo@usc.edu (C.-C.J.K.); 3 Glickman Urological Institute, Cleveland Clinic, Cleaveland, OH 44125, USA; helle246@umn.edu (N.H.); weightc@ccf.org (C.J.W.); 4 Cleveland Clinic Lerner, School of Medicine, College of Medicine of Case, Western Reserve University, Cleveland, OH 44106, USA; 5 Alfred E Mann Department of Biomedical Engineering, University of Southern California, Los Angeles, CA 90089, USA; 6 Institute of Urology, University of Southern California, Los Angeles, CA 90033, USA; 7 Department of Radiology, Los Angeles General Medical Center, Los Angeles, CA 90033, USA

**Keywords:** artificial intelligence, medical image segmentation, sustainability

## Abstract

As artificial intelligence advances in medical image analysis, its environmental impact remains largely overlooked. This study analyzes the energy demands of AI workflows for medical image segmentation using the popular Kidney Tumor Segmentation-2019 (KiTS-19) dataset. It examines how training and inference differ in energy consumption, focusing on factors that influence resource usage, such as computational complexity, memory access, and I/O operations. To address these aspects, we evaluated three variants of convolution—Standard Convolution, Depthwise Convolution, and Group Convolution—combined with optimization techniques such as Mixed Precision and Gradient Accumulation. While training is energy-intensive, the recurring nature of inference often results in significantly higher cumulative energy consumption over a model’s life cycle. Depthwise Convolution with Mixed Precision achieves the lowest energy consumption during training while maintaining strong performance, making it the most energy-efficient configuration among those tested. In contrast, Group Convolution fails to achieve energy efficiency due to significant input/output overhead. These findings emphasize the need for GPU-centric strategies and energy-conscious AI practices, offering actionable guidance for designing scalable, sustainable innovation in medical image analysis.

## 1. Introduction

AI-driven medical image analysis has made remarkable progress, revolutionizing diagnoses and treatment planning. Convolutional Neural Networks (CNNs) and other deep learning models have enabled significant advancements in tasks such as segmentation and classification, leading to improved diagnostic accuracy [1]. These innovations streamline workflows and enhance patient outcomes by allowing more precise and timely diagnoses. However, the increased use of AI technology in healthcare has raised concerns about its environmental impact [2,3,4,5]. The computational capacity for training cutting-edge models frequently depends on energy-demanding GPUs, resulting in substantial carbon emissions. Moreover, the recurrent inference tasks in clinical settings exacerbate the total energy consumption over a model’s lifecycle. As the healthcare sector increasingly integrates AI, understanding and mitigating the environmental footprint of these technologies is critical. Balancing AI’s potential to revolutionize medical imaging with sustainable practices is necessary to ensure that these advancements do not come at the cost of environmental degradation. This challenge calls for a concerted effort to develop energy-efficient AI models, optimize workflows, and adopt green computing practices in medical imaging applications [6].

With an estimated 20 million new cancer cases annually [7], and most patients requiring at least one imaging study for diagnosis, staging, or treatment planning, the cumulative energy usage for these analyses amounts to approximately 28,540 kWh per year. To put this into perspective, this is equivalent to the annual energy consumption of around 2–3 average USA. households or running a refrigerator non-stop for nearly 81 years. This significant energy demand highlights the hidden environmental cost of image analysis in modern healthcare. As imaging technologies become increasingly central to precision medicine and cancer care, the number of imaging studies performed is expected to grow exponentially. Without targeted efforts to improve energy efficiency, the environmental impact of these essential diagnostic tools will continue to escalate. Moreover, this calculation considers only medical imaging; if other areas of medicine, such as drug discovery or genomic analysis, are considered, the energy consumption costs would grow exponentially, emphasizing the critical need for energy-conscious advancements across all domains of healthcare. This underscores the importance of developing optimized computational pipelines, energy-efficient hardware, and more sustainable approaches to large-scale image analysis to balance the critical benefits of imaging with the need to reduce its carbon footprint.

Several studies have provided valuable insights into energy-efficient AI design [8]. A study conducted by Yang et al. [9] introduced a methodology to estimate DNN energy consumption based on architecture, sparsity, and bit-width, emphasizing that data movement dominated energy costs over computation. By leveraging memory hierarchy optimization and analyzing layer-wise energy, the approach provided insights into energy-efficient DNN design. It also demonstrated the trade-offs between accuracy and energy consumption and offered practical tools like energy-aware pruning to optimize performance. An interesting study by Naser compares simple and exotic machine learning (ML) algorithms, assessing their predictive performance, model size, energy consumption, and carbon emissions [10]. It explores the impact of algorithm architecture, programming language, dataset size, and feature selection on accuracy and energy efficiency. Key findings include the potential for 23–99% reductions in energy consumption and emissions with simpler models and reduced-order strategies without compromising accuracy. XGBoost demonstrated the highest predictivity, while logistic regression had the lowest energy consumption. Hajabdollahi et al. [11] presented a method to simplify Convolutional Neural Networks (CNNs) for efficient skin lesion segmentation, targeting portable medical devices with limited resources. Their study introduced a color channel pruning technique to select the most informative channels, reducing network complexity while maintaining high segmentation accuracy. Experimental results on dermoscopic and non-dermoscopic datasets demonstrated that pruning significantly reduced computational and memory requirements without substantial loss of accuracy. The findings highlighted the potential of this approach for real-time, resource-constrained medical applications. Wen and Gregg [12] explored additional strategies for compressing Convolutional Neural Networks (CNNs) by leveraging redundant patterns in weight tensors that remain after pruning and quantization. Their study introduced a Shared Block Sparse Row (SBSR) format to compact weight data by identifying and referencing repeated weight blocks, achieving superior compaction ratios beyond existing techniques.

A ground-breaking analysis by the researchers at OpenAI revealed the staggering growth in computational demands for training large AI models [13]. Their research demonstrated that the compute used in training state-of-the-art AI systems has been doubling every 3.4 months since 2012—a far more aggressive growth rate than Moore’s Law, which predicts the doubling of transistors in a chip roughly every two years. Over just six years, this exponential increase led to a 300,000× growth in the computational power required to train these models. This rapid escalation underscores the immense resource demands of cutting-edge AI research, driven by increasingly complex architectures and larger datasets. While this trend has enabled breakthroughs in areas like natural language processing and computer vision, it also raises critical questions about sustainability, energy efficiency, and the environmental impact of training such massive models. Addressing these challenges will be essential to balance AI advancements with responsible and sustainable computational practices.

While significant attention has been directed toward the high computational demands of training AI models, the cumulative energy usage from repeated inference on medical data in clinical practice remains an underexplored area. To address this gap, we demonstrate our analysis of the KiTS dataset [14]. We provide a detailed quantitative evaluation of energy consumption across different convolution types and optimization techniques during the training and inference phases. These findings offer practical insights for reducing the environmental footprint of AI models, guiding developers in designing efficient workflows that balance computational performance with sustainability, ultimately contributing to greener, scalable solutions.

## 2. Methodology

**Figure 1:**
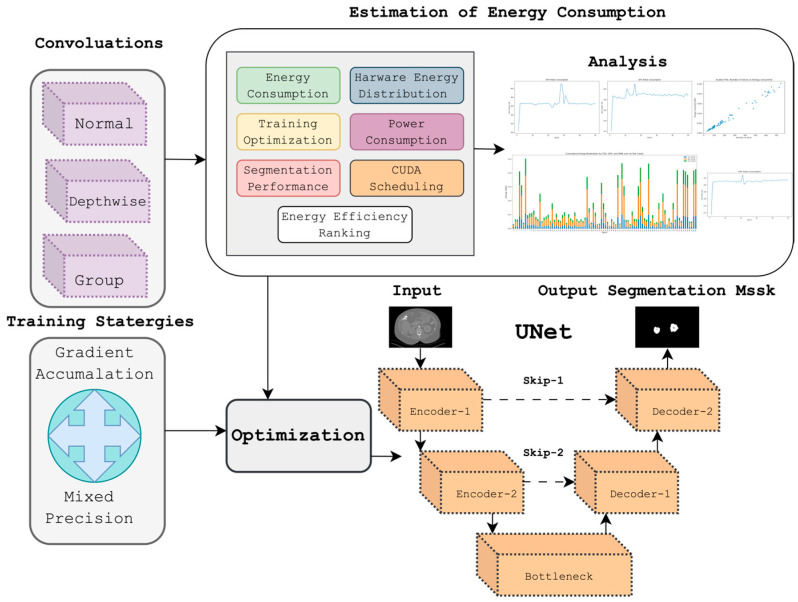
Overview of the study focusing on energy consumption in medical image segmentation.

A comprehensive methodology was employed to evaluate the energy-performance trade-offs: data pre-processing, convolutional variants, optimization techniques, the experimental setup, energy measurement, and evaluation metrics. Data pre-processing includes data loading, normalization, and slice extraction. Convolutional variants cover architectures such as Standard, Depthwise [15], and Group Convolutions [16], evaluated for computational efficiency. Optimization techniques focus on Mixed Precision [17] and Gradient Accumulation [18,19] to optimize memory and energy usage: the experimental setup details both the training and testing phases for model evaluation. Energy measurement involves monitoring power consumption during training and testing using key metrics. Finally, evaluation metrics assess model performance based on segmentation accuracy and energy efficiency. Figure 1 represents the schematic representation of the proposed study.

### 2.1. Pre-Processing

The preprocessing pipeline for the KiTS dataset comprised several critical steps to optimize the data for segmentation tasks. Voxel intensities were normalized to the [0, 1] range to ensure consistency and reduce scanner-related variability. The images were then resampled to a uniform voxel spacing to standardize spatial resolution across cases. Finally, 3D volumes were decomposed into 2D axial slices to reduce computational complexity and facilitate model training.

### 2.2. Convolutional Variants

To analyze energy-performance trade-offs, three convolutional variants with varying computational complexities were selected: (1) Standard convolution—used as a baseline, this architecture relies on conventional convolutions for feature extraction, providing strong performance but at a high computational cost; (2) Depthwise convolution—selected for its ability to reduce computational complexity by separating spatial and depthwise operations. Unlike standard convolution, which applies a single filter across all input channels, Depthwise Convolution processes each channel independently with depthwise filters, followed by a pointwise convolution to combine outputs. This separation reduces the number of parameters and floating-point operations, making it a computationally efficient alternative; (3) Group convolution—chosen for its ability to reduce computational complexity by dividing input channels into smaller groups, applying convolutions independently within each group. While this reduces the number of parameters and floating-point operations, it also introduces additional data movement overhead. Traditional machine learning models such as XGBoost or Random Forests were not considered, as they are not directly applicable to pixel-wise semantic segmentation tasks on image data. These models operate on structured tabular inputs and require extensive handcrafted feature extraction, which would distort the problem formulation and invalidate direct energy-performance comparisons with CNN-based methods. Hence, our evaluation focuses solely on convolutional variants within the U-Net framework to ensure consistency and relevance to end-to-end medical image segmentation.

### 2.3. Optimization Techniques

Two key techniques were applied to explore the role of training optimizations in energy efficiency, namely (1) Mixed Precision and (2) Gradient Accumulation. Mixed Precision enhances computational efficiency by utilizing FP16 (16-bit floating-point precision) for most operations while retaining FP32 (32-bit precision) for critical calculations such as gradient accumulation and loss scaling [20]. It reduces memory consumption and bandwidth usage, enabling larger batch sizes and faster computation without sacrificing numerical stability. Mixed Precision Training was applied across all architectures in this study to quantify its impact on energy savings and performance. Its ability to leverage hardware features like NVIDIA’s Tensor Cores makes it particularly suited for energy-efficient workflows, reducing training time and potentially lowering the carbon footprint of AI workflows in medical image analysis. Gradient Accumulation is an optimization technique that simulates larger batch sizes by accumulating gradients over multiple smaller mini-batches before performing a single weight update, helping avoid GPU memory limitations. By applying Gradient Accumulation, this study evaluated whether it results in lower energy consumption during training by reducing the frequency of weight updates and balancing memory usage. Both optimization strategies were tested individually and in combination to assess their cumulative impact on energy consumption and efficiency in medical image analysis workflows.

### 2.4. Dataset Description

**Figure 2:**
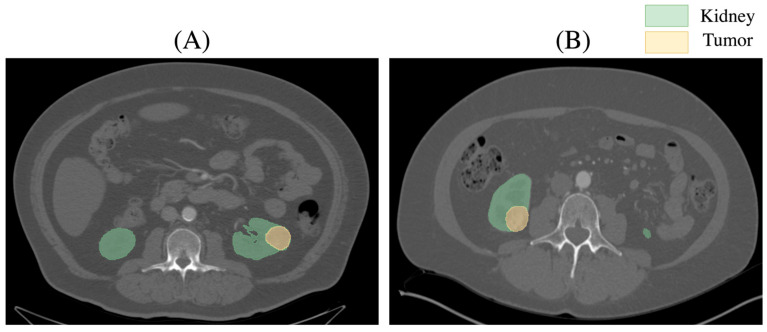
Representative axial CT slice from the KiTS dataset depicting a renal tumor and surrounding kidney anatomy. The tumor is located at the lower pole of the left kidney in (**A**) and at the lower pole of the right kidney in (**B**).

The Kidney Tumor Segmentation-2019 dataset is a widely used benchmark for developing and evaluating algorithms in medical image analysis. It consists of annotated Computed Tomography (CT) scans of patients with kidney tumors, providing ground truth segmentations for three classes: background, kidney, and tumor. This dataset is designed to facilitate research on automated segmentation methods, with the goal of improving the accuracy of detecting and delineating renal masses. The KiTS dataset poses significant challenges due to variations in tumor size, shape, and location. Figure 2 shows axial CT slices from the KiTS dataset with segmented kidneys (green) and tumors.

### 2.5. Experimental Setup

A series of experiments was designed to comprehensively evaluate the energy consumption and performance of various model configurations, spanning different convolutional variants and optimization techniques (Table 1). The experiments aimed to analyze the energy-performance trade-offs under varying conditions, focusing on both training and testing phases.

All models in our study are based on a standardized 2D U-Net encoder–decoder architecture with skip connections and five resolution levels, using feature channels [32, 64, 128, 256, 512]. The baseline model employs conventional convolutional blocks: each encoder stage consists of two 3 × 3 convolutions followed by batch normalization, Leaky ReLU activation, and dropout (increasing progressively from 0.05 to 0.5), with 2 × 2 max pooling for spatial downsampling. Decoder stages use transposed convolutions for upsampling, followed by the same dual-convolutional structure after concatenation with the corresponding encoder skip connections. The output layer is a 3 × 3 convolution producing three-class segmentation maps. To examine how the effect of convolutional design impacts performance and energy efficiency, we implemented two architectural variants by replacing standard convolutions with (i) depthwise separable convolutions, which decompose each 3 × 3 operation into a depthwise convolution per channel followed by a 1 × 1 pointwise projection, and (ii) group convolutions, with the number of groups dynamically determined (up to 8) based on input channel divisibility to encourage structured sparsity. All three model variants preserve identical architectural depth, feature dimensionality, and training hyperparameters. Kaiming normal initialization was applied to all convolution layers. The input data comprised 2D grayscale CT slices of size 512 × 512, with output predictions represented as soft segmentation masks for three semantic classes. To ensure strict reproducibility, we fixed the random seed to 42 across Python 3.9.19, Numpy 1.26.4, and PyTorch 2.4.0 (CPU and CUDA 12.2), ensuring consistent weight initialization, data shuffling, and dropout behavior. Furthermore, PyTorch’s deterministic mode was enabled while disabling cuDNN benchmarking to prevent backend variability due to non-deterministic algorithm selection on GPUs. This controlled setup enables a fair, repeatable comparison of architectural variants, isolating the impact of convolutional design choices on segmentation performance and computational efficiency.

### 2.6. Training and Testing Phases

In the Training Phase, slices with mask values of 1 or 2 (representing kidney and tumor regions) were selected for training. In contrast, slices with a mask value of 0 (background) were excluded to reduce computational overhead and improve training efficiency. The training process utilized a U-Net model with a batch size of 8, running for 50 epochs, chosen for its proven effectiveness in medical image segmentation. Energy consumption during training was monitored using the CodeCarbon [21], which tracked CPU, GPU, and RAM usage. This allowed for detailed insights into energy consumption across each epoch, facilitating a granular resource usage analysis throughout the training process.

In the Testing Phase, all slices were included to ensure a comprehensive evaluation of the model’s performance. Energy consumption for each subject case was monitored using the CodeCarbon library, which enabled an analysis of the energy demands for individual patient cases during inference, providing insights into resource usage and energy efficiency during the testing phase.

### 2.7. Energy Measurement

Energy metrics for this study were estimated using the CodeCarbon library, which provides an approximate measure of power usage and associated CO_2_ emissions. CodeCarbon operates by leveraging pre-defined hardware efficiency metrics, such as the power consumption of GPUs, CPUs, and other components, in conjunction with regional carbon intensity values. The analysis in this study was divided into two phases: training and testing, with the energy consumption patterns and CO_2_ estimates presented for each phase.

Training Analysis: In training, key metrics were extracted from CodeCarbon to assess energy consumption throughout the training phase. These metrics include: (1) Epoch—which tracks energy consumption and emissions for each training iteration, allowing for temporal analysis of resource usage; (2) Duration—which measures the time taken for each epoch, providing insights into the correlation between time and energy consumption; (3) Emissions—representing the carbon emissions (in kgCO_2_eq) generated during each epoch, serving as an indicator of environmental impact; (4) Power Metrics—monitoring hardware-specific power usage, including CPU Power, GPU Power, and RAM Power (in watts), offering insights into the energy demands of each hardware component; (5) Energy Metrics—capturing the total energy consumed by each hardware component, including CPU Energy, GPU Energy, and RAM Energy (in kWh), to provide a breakdown of resource usage at the component level; and (6) Total Energy Consumption—which records the overall energy consumption (in kWh) for each epoch, offering a holistic view of resource usage during training. The relationship between the number of epochs and energy consumption is provided in the Supplementary Material (Figure S2).

Testing Analysis: Energy consumption during inference was analyzed using key metrics extracted from the CodeCarbon, providing a comprehensive view of each test case’s resource usage and environmental impact. These metrics included: (1) Number of slices per case or subject—which correlates directly with processing requirements and energy consumption, as more slices generally lead to higher resource usage; (2) Duration—which measures the time taken per test case, helping to correlate energy consumption with processing time; (3) Emissions—which represents the carbon emissions (in kgCO_2_eq) generated for each case, serving as a key indicator of environmental impact; (4) Power Metrics—monitoring component-specific power consumption, including CPU Power, GPU Power, and RAM Power (in watts), offering insights into energy demands at the hardware level; (5) Energy Metrics—capturing the total energy consumed by each hardware component, including CPU Energy, GPU Energy, and RAM Energy (in kWh), providing a breakdown of cumulative energy usage; and (6) Total Energy Consumption—which records the overall energy consumption (in kWh) for each test case, offering a holistic view of resource usage during inference. The relationship between the above-mentioned parameters and the energy consumption is provided in the Supplementary Material (Figure S1).

## 3. Results and Discussion

This section provides details about 1. Energy Consumption across Convolution Types, focusing on how architectural choices impact overall energy usage. Additionally, it investigates 2. Impact of Training Optimizations to assess their role in reducing energy demands, improving computational efficiency, and enhancing model stability. The study further analyzes 3. Energy Distribution among Hardware Components, highlighting the contributions of GPU, RAM, and CPU to the total energy footprint, followed by 4. Segmentation Performance across all Configurations and 5. GPU Power Consumption across Training Experiments. This follows an analysis of 6. Per-slice energy consumption analysis during inference. Finally, 7. Energy efficiency rankings provide a comparative evaluation of the most and least energy-efficient architectural configurations. This analysis comprehensively explains energy efficiency trends and their broader implications for sustainable workflows.

### 3.1. Energy Consumption Across Convolution Types

The study highlights significant variations in energy consumption across different convolutional architectures, revealing the complex relationship between model design, parameters, and energy efficiency. Detailed comparisons of Standard, Depthwise, and Group Convolutions underscore the importance of architectural choices in influencing energy demands. This analysis considers both the training and testing phases, offering insights into how each architecture performs in terms of computational load, scalability, and practical deployment.

Training Phase: During training, energy consumption varied across convolution types, reflecting trade-offs between parameter reduction and computational overhead. Standard Convolution, used as the baseline, consumed 5.209 kWh (Table 2), with the GPU contributing 1.675 kWh, CPU 0.456 kWh, and RAM 0.522 kWh, highlighting the cost of convolutions without parameter-efficient strategies. Depthwise Convolution, which reduced parameters from 7.2 M to 1 M, consumed 5.770 kWh. Although more lightweight, its fragmented per-channel operations increased GPU energy to 2.005 kWh, with CPU and RAM usage at 0.434 and 0.496 kWh, respectively, driven by frequent memory access. Group Convolution, despite lowering parameters to 1.2 M, showed the highest consumption at 6.103 kWh, due to memory and I/O inefficiencies. RAM energy peaked at 0.600 kWh, GPU at 1.974 kWh. At the per-epoch level (Table 3), Standard Convolution averaged 0.1042 kWh, improved slightly with Mixed Precision (0.1020 kWh), while Gradient Accumulation raised usage to 0.1161 kWh due to extended epoch durations. Depthwise Convolution achieved the lowest per-epoch energy at 0.0920 kWh under Mixed Precision, but increased to 0.1048 kWh with Gradient Accumulation. Group Convolution consistently remained high across configurations (~0.122 kWh/epoch), with elevated GPU demands. Despite reducing parameters, both Depthwise and Group Convolutions incurred overheads that diminished their computational efficiency.

Testing Phase: During inference, Standard Convolution served as the baseline, consuming 0.001427 kWh per test case (Table 4). GPU usage dominated at 0.000777 kWh, with CPU and RAM contributing 0.000303 kWh and 0.000347 kWh, respectively. Average GPU power was 211.16 W. As shown in Supplementary Figure S2, energy usage increased linearly with slice count, and positively correlated with inference duration, as each slice undergoes the same processing regardless of complexity. Slices were considered more complex when tumors (Class 2) were embedded within kidneys (Class 1), adding segmentation difficulty and increasing resource demand. Depthwise Convolution consumed more energy (0.001686 kWh per case), with GPU usage rising to 0.001051 kWh and average GPU power spiking to 291.38 W. Though inference time slightly dropped to 12.95 min, fragmented per-channel operations required more frequent memory access, amplifying GPU load especially on complex cases. Group Convolution averaged 0.001479 kWh per case, peaking at 0.005463 kWh in outliers. GPU energy was 0.000808 kWh, while CPU and RAM added 0.000313 kWh and 0.000358 kWh, respectively. Inference time was longest at 13.67 min, reflecting overhead from grouped-channel operations and memory inefficiencies. GPU power stayed high at 212.58 W. Across all configurations, Standard Convolution proved the most energy-efficient for testing, with the lowest GPU demands. Depthwise Convolution, despite reduced inference time, incurred higher GPU energy due to fragmented processing. Group Convolution was the least efficient, with longer durations and elevated I/O overhead. Test cases with more slices or longer runtimes consistently drove up energy usage, with GPU consumption remaining the primary contributor.

While Depthwise Convolution significantly reduces parameter count, it exhibits the highest GPU energy consumption during inference. This seemingly counterintuitive outcome arises from the fragmented nature of depthwise operations. Unlike Standard Convolution, which applies dense filters across all input channels in a single fused operation, Depthwise Convolution processes each channel independently, resulting in a large number of lightweight kernel launches. These fragmented procedures lead to low arithmetic intensity, meaning relatively few computations per memory access and force the GPU to perform more frequent memory fetches, context switches, and kernel invocations. This overhead degrades memory bandwidth efficiency and stalls GPU compute pipelines, particularly on architectures optimized for high-throughput matrix operations. Furthermore, inference lacks backpropagation, so the GPU has no opportunity to amortize these memory costs over gradient computations. As a result, although Depthwise Convolution theoretically lowers compute cost and parameter footprint, it incurs greater instantaneous power draw and longer total runtime during inference. This inefficiency was evident in our results: Depthwise Convolution recorded the highest per-case GPU energy (0.001051 kWh) and peak power draw (291.38 W), even exceeding the denser Standard Convolution, which benefited from contiguous memory access and hardware-accelerated cuDNN support. These findings highlight that architectural sparsity alone does not guarantee runtime energy efficiency and underscore the importance of aligning model structure with hardware execution profiles.

### 3.2. Impact of Training Optimizations

The study thoroughly evaluated the impact of Mixed Precision and Gradient Accumulation on energy efficiency across different convolutional configurations, revealing distinct trade-offs between energy consumption, computational efficiency, and model stability.

Mixed Precision: Mixed Precision consistently demonstrated its effectiveness in reducing energy consumption by using FP16 computations for most operations while maintaining model performance. Across all convolution types, it led to measurable reductions in both energy usage and epoch time compared to unoptimized setups. In Standard Convolution, energy dropped from 5.209 kWh to 5.099 kWh (2.1% reduction), and average epoch time decreased from 782.3 s to 674.5 s, showing clear gains in energy efficiency (Table 2 and Table 3). Depthwise Convolution saw the most substantial benefit: total energy fell from 5.770 kWh to 4.601 kWh (20.3% decrease). GPU energy dropped from 2.005 to 1.559 kWh, CPU from 0.434 to 0.365 kWh, and RAM from 0.496 to 0.418 kWh. Per-epoch energy dropped from 0.1154 to 0.0920 kWh, with epoch time reduced from 745.5 s to 626.5 s, highlighting how precision reduction complemented its lightweight architecture. Group Convolution, however, showed minimal improvements. Energy consumption dropped only 0.57% (from 6.103 to 6.068 kWh), with mean GPU power reduced slightly from 308.24 W to 299.38 W. Per-epoch energy stayed nearly flat (~0.1214 kWh), with negligible impact on training duration, largely due to persistent I/O and memory overheads from grouped-channel operations. In summary, Mixed Precision led to consistent energy savings, especially in Depthwise Convolution, where reduced precision synergized with fewer parameters. However, architectures like Group Convolution saw limited benefit, emphasizing that precision gains can be masked by inefficient memory patterns.

Gradient Accumulation: Gradient Accumulation, aimed at enabling larger effective batch sizes in memory-constrained settings, led to increased energy consumption across most configurations due to prolonged training durations. For Standard Convolution, total energy rose from 5.209 to 5.802 kWh (an 11.4% increase), with mean epoch duration increasing from 782.3 s to 889.6 s. GPU energy also climbed to 1.848 kWh, reflecting the overhead of aggregating gradients before weight updates (Table 2 and Table 3). In Depthwise Convolution, total energy decreased slightly from 5.770 to 5.237 kWh, but this came with a cost: mean training time extended to 5.8 h, up from 4.4 h under Mixed Precision. Despite the energy drop, the time-efficiency trade-off was significant. Group Convolution saw negligible gains. Energy dropped marginally from 6.103 to 6.090 kWh, but mean epoch duration hit 6.43 h (23,138.94 s)—the longest of all configurations. GPU energy stayed high at 1.992 kWh, while memory and I/O bottlenecks persisted (Table 2 and Table 3). These results show that while Gradient Accumulation aids model stability and scaling, it introduces a steep energy and time overhead. For compute-heavy or fragmented architectures like Depthwise and Group Convolutions, the benefits are outweighed by the increased runtime and power demands, making it less suitable for energy-conscious workflows. Despite its higher energy footprint, Gradient Accumulation remains a practical and often necessary strategy in memory-constrained environments where large batch sizes cannot be accommodated. In clinical or federated setups using mid-range GPUs, GA enables stable training by simulating larger effective batches. Additionally, it can help smooth noisy gradients in small-batch slice-wise segmentation tasks, improving convergence behavior. While our results show that GA increases training time and total energy, its use may still be justified in scenarios where hardware constraints or dataset-specific challenges necessitate more stable gradient aggregation.

### 3.3. Energy Distribution Among Hardware Components

Training Phase: As shown in Supplementary Figure S2, energy consumption during training is dominated by the GPU, with RAM and CPU contributing secondary but notable amounts. This trend holds across all convolution types, though the exact proportions vary based on architecture. In Standard Convolution, the GPU accounted for 1.675 kWh, or 32.1% of the total 5.209 kWh, with RAM and CPU contributing 0.522 kWh (10%) and 0.456 kWh (8.8%), respectively (Table 2). On a per-epoch basis, GPU energy averaged 0.0658 kWh, while CPU and RAM contributed 0.0179 and 0.0205 kWh, bringing the total to 0.1042 kWh per epoch (Table 3). Cumulative energy steadily increased with training duration. In Depthwise Convolution, GPU energy rose to 2.005 kWh, forming 34.7% of the total 5.770 kWh. CPU and RAM added 0.434 and 0.496 kWh, respectively, reflecting the increased demand from fragmented channel-wise operations. Per-epoch GPU energy rose to 0.0788 kWh, while CPU and RAM were 0.0171 and 0.0195 kWh. Total per-epoch energy was 0.1154 kWh, improving to 0.0920 kWh with Mixed Precision (Table 3). Group Convolution had GPU usage of 1.974 kWh (32.3% of total 6.103 kWh), with RAM and CPU at 0.600 kWh (9.8%) and 0.525 kWh (8.6%). Its per-epoch GPU energy averaged 0.0777 kWh, while CPU and RAM consumed 0.0207 and 0.0237 kWh, for a total of 0.1221 kWh per epoch, consistently the highest among all models (Table 3). These results reaffirm that while GPU is the main energy consumer, the architectural structure significantly affects hardware distribution. Depthwise Convolution increases GPU demand due to fragmented operations, while Group Convolution introduces greater RAM usage and I/O overhead, undermining its energy efficiency. Despite reducing parameters, both Depthwise and Group Convolutions incurred overheads that diminished their energy efficiency. These findings reflect a broader trade-off in modern model design: while such convolutions significantly lower the number of operations and parameters, they often introduce fragmented memory access patterns that increase latency and power draw, particularly on GPU architectures sensitive to memory throughput and access granularity. Although these operations offer theoretical reductions in computation, the practical energy gains may be limited unless paired with compiler-level or hardware-aware optimizations. Strategies to improve memory efficiency include operator fusion (e.g., combining depthwise and pointwise layers to minimize kernel launch overhead), tensor layout reordering for better cache locality, and adaptive group sizing to balance parameter savings with memory bandwidth efficiency. Future work should evaluate these implementation-level strategies to fully leverage the potential energy savings of lightweight convolutional designs.

**Figure 3:**
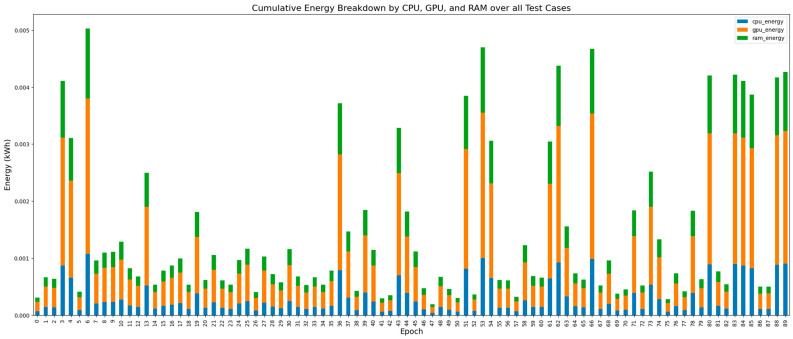
Energy breakdown by CPU, GPU, and RAM for each test case during testing for Standard Convolution, Highlighting GPU’s dominant energy consumption and consistent trends across all test cases.

Testing Phase: As illustrated in Figure 3, energy distribution during testing is dominated by the GPU, with RAM and CPU contributing secondary loads. This pattern holds across all convolution types, though the exact breakdown varies. For Standard Convolution, GPU energy averaged 0.000777 kWh, comprising 54.5% of the total 0.001427 kWh (Table 4). RAM and CPU contributed 0.000347 kWh (24.3%) and 0.000303 kWh (21.2%), respectively. These values reflect the GPU’s role in executing forward passes, while RAM and CPU handled data transfer and memory operations. In Depthwise Convolution, GPU consumption increased to 0.001051 kWh, making up 62.3% of the total 0.001686 kWh. RAM and CPU contributed 0.000392 kWh (23.3%) and 0.000243 kWh (14.4%). The high GPU power draw (291.38 W) highlights the cost of fragmented channel-wise processing and frequent memory access. Group Convolution showed GPU energy at 0.000808 kWh (54.6% of 0.001479 kWh), with RAM and CPU at 0.000358 (24.2%) and 0.000313 kWh (21.2%). Despite fewer parameters, energy inefficiencies persisted due to grouped-channel I/O overhead, with GPU power averaging 212.58 W. Across all models, GPU energy consistently ranged from 54–62% of the total test-time consumption (Table 4), reaffirming its role as the dominant driver. RAM energy remained stable due to activation transfers, while CPU usage varied slightly. In summary, Depthwise Convolution demanded the highest GPU power during inference due to fragmented operations, and Group Convolution suffered from inefficiencies tied to memory access and I/O. Standard Convolution maintained the most balanced and efficient hardware distribution.

### 3.4. Segmentation Performance Across All Configurations

The segmentation results for Standard, Depthwise, and Group Convolutions (Table 5) reveal distinct performance trends across classes, highlighting trade-offs between architectural choices and training optimizations. For Class 0 (Background), all configurations achieved near-perfect results, with Dice scores ≈ 0.9992 and precision >0.9996, showing minimal variation across setups. In Class 1 (Kidney), Standard Convolution with Mixed Precision delivered the highest Dice score (0.9055). Depthwise Convolution performed slightly worse at 0.8988, and Group Convolution was consistently lower, peaking at 0.8961. Although differences were minor, Group Convolution showed a clear downward trend in precision and recall. Class 2 (Tumor) performance showed the most variance. Standard Convolution with Mixed Precision again led with a Dice score of 0.7332. Depthwise dropped to 0.6728, and Group Convolution reached a modest 0.6755. Precision and recall were also lower for Depthwise and Group, with Group’s precision at 0.8446, compared to 0.8720 in Standard. Overall, Mixed Precision improved segmentation across all architectures, but Standard Convolution maintained the best and most stable performance, particularly for complex regions like tumors. In contrast, Depthwise and Group Convolutions traded segmentation accuracy for parameter and computational efficiency, especially in classifying finer structures.

For a more detailed, per-class breakdown of segmentation performance across architectures, readers are referred to the Supplementary Material (Section S1.3). Supplementary Figures S6 and S7 provide grouped bar plots and extended analysis specifically for Kidney (Class 1) and Tumor (Class 2), highlighting nuanced performance trends, such as the high sensitivity of Standard Convolution U-Nets and the recall limitations in depthwise and grouped variants when segmenting sparse tumor regions. These visualizations complement Table 5 by providing deeper insights into class-specific segmentation dynamics under different architectural and optimization configurations.

### 3.5. GPU Power Consumption Across Training Experiments

Understanding GPU power consumption during training is crucial for evaluating energy efficiency and computational performance across different neural network architectures. This section analyses power usage patterns across Standard, Depthwise, and Group Convolutions under various training configurations, including unoptimized setups, Mixed Precision, and Gradient Accumulation. These insights highlight the interplay between architectural choices, optimization techniques, and energy demands, providing a comprehensive understanding of GPU power behavior during training.

**Figure 4:**
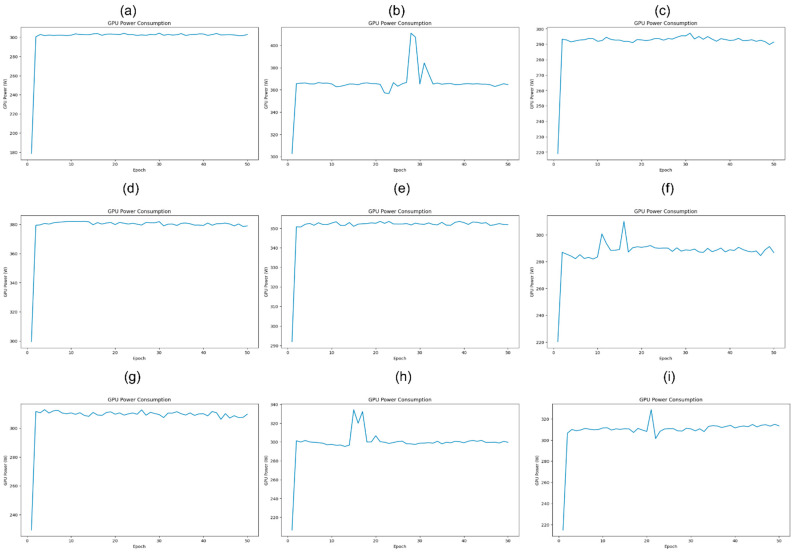
GPU power consumption across various training configurations. (**a**) Standard Convolution during training without optimization; (**b**) Standard Convolution during training with Mixed Precision; (**c**) Standard Convolution during training with Gradient Accumulation; (**d**) Depthwise separable convolution during training without Optimization; (**e**) Depthwise separable convolution during training with Mixed Precision; (**f**) Depthwise separable convolution during training with Gradient Accumulation; (**g**) Group Convolution during training without Optimization; (**h**) Group Convolution during training with Mixed Precision; (**i**) Group Convolution during training with Gradient Accumulation.

Standard Convolution: During training, GPU power patterns for Standard Convolution varied across optimization techniques (Figure 4). In the unoptimized setup (Figure 4a), power spiked from ~180 W to over 300 W during the first epoch, a ramp-up phase as the GPU transitioned from idle to full compute load. Afterward, power stabilized around 300 W, showing consistent load and efficient utilization throughout training. With Mixed Precision (Figure 4b), a similar ramp-up occurred, but power stabilized at a higher range (350–380 W). Intermittent spikes above 400 W were observed, likely due to occasional operations needing higher precision or memory access. Despite these outliers, the system returned quickly to a steady state, showing robustness under fluctuating loads. Under Gradient Accumulation (Figure 4c), GPU power initially rose quickly, then leveled off around 290 W. Unlike other configurations, this setup showed no significant spikes, the workload distribution from smaller mini-batches smoothed power demands, resulting in a highly stable power profile across epochs.

Depthwise Separable Convolution: In the unoptimized setup, GPU power ramped up from ~300 W to 380 W during the first epoch and then held a stable plateau near 380 W for the remainder of training. This steady usage reflects the predictable behavior of Depthwise operations, where independent per-channel computations lead to high memory access overhead but minimal power fluctuation. With Mixed Precision (Figure 4e), the ramp-up began lower, from 290 W to just over 350 W, after which power remained consistently above 350 W. The absence of spikes demonstrates how reduced-precision computations smooth out GPU demands, offering energy savings and workload stability. In contrast, Gradient Accumulation (Figure 4f) showed a different pattern. Power ramped from ~220 W to 280 W, followed by spikes exceeding 300 W in early epochs. Once past this phase, consumption settled around 280 W, with small variations. This reflects how gradient accumulation spreads the workload over time, reducing peak demand but occasionally triggering short bursts of intensity due to memory and sync overhead.

Group Convolution: The GPU power during Group Convolution training exhibited distinct patterns across configurations (Figure 4). In the unoptimized setup (Figure 4g), a sharp spike in power occurred during the first epoch as the model initialized computation and data handling. Power then stabilized around 300 W, with only minor fluctuations, reflecting consistent GPU load and efficient utilization across epochs.

Precision (Figure 4h), the initial ramp-up was similar, but the first 15 epochs showed more pronounced fluctuations, with peaks exceeding 320 W. These early spikes reflect the GPU adjusting to FP16/FP32 operations. Once stabilized, power hovered near 300 W, indicating that Mixed Precision maintained an efficient but slightly more variable power profile compared to the unoptimized baseline. Training with Gradient Accumulation (Figure 4i) also began with a high initial surge, followed by stable power at ~300 W throughout most epochs. However, an isolated spike above 320 W appeared—likely caused by asynchronous data loading or delayed gradient synchronization, briefly overloading the GPU. After this, power returned to baseline, demonstrating generally effective workload management with occasional sync-induced disruptions. The power spikes observed during training, particularly under Mixed Precision and Gradient Accumulation, are primarily due to CUDA scheduling inefficiencies, dynamic kernel switching, and synchronization delays. These fluctuations are especially pronounced in fragmented architectures like Depthwise and Group Convolutions. A detailed breakdown of these power spike patterns and scheduling behavior is provided in the Supplementary Material (Section S1.1).

Importantly, despite these GPU power fluctuations, we observed no adverse effects on training stability, convergence behavior, or segmentation accuracy; further details are provided in the Supplementary Material (Section S1.2). All Mixed Precision configurations achieved stable loss trajectories and successful convergence, with no signs of numerical instability such as gradient explosion or divergence. Segmentation metrics presented in Table 5 confirm that accuracy remained consistent or improved under Mixed Precision training—particularly for Standard Convolution, which achieved the highest Dice score for tumor regions (0.7332). This indicates that the spikes reflect execution-level dynamics and do not impair the optimization process or final model quality.

### 3.6. Per-Slice Energy Consumption Analysis During Testing

As shown in Supplementary Figure S3, Standard Convolution exhibited consistent and efficient per-slice energy usage, averaging 0.00000192 kWh, with minimal fluctuation across workloads. The GPU remained the primary contributor, followed by RAM and CPU. Energy usage was proportional to the number of slices, not their anatomical complexity, due to a uniform computational pipeline applied to each slice. Depthwise Convolution averaged 0.00000215 kWh per slice, with greater variability arising from fragmented channel-wise operations and increased memory access. Group Convolution consumed the most energy per slice (0.00000232 kWh), with spikes caused by irregular memory access and I/O overhead. These results underscore that while all models follow identical processing per slice, architectural fragmentation negatively impacts energy efficiency, with Standard Convolution being the most stable and energy-efficient option for slice-based inference.

### 3.7. Energy Efficiency Ranking

Training Phase: The energy efficiency during training varied significantly across configurations. Depthwise Convolution with Mixed Precision was the most efficient, consuming 4.601 kWh, a 20.3% reduction from its unoptimized baseline (5.770 kWh). This setup reduced GPU energy to 1.559 kWh, CPU to 0.365 kWh, and RAM to 0.418 kWh, leveraging the synergy between channel-wise operations and FP16 precision. Standard Convolution with Mixed Precision ranked second, with energy use reduced to 5.099 kWh from 5.209 kWh (2.1%). In contrast, Gradient Accumulation increased energy usage to 5.802 kWh due to extended training durations. Group Convolution consistently ranked lowest in energy efficiency. Its unoptimized setup consumed 6.103 kWh, with Mixed Precision providing minimal benefit (6.068 kWh, 0.57% reduction) and Gradient Accumulation showing similarly limited improvement (6.090 kWh). GPU energy remained high at ~1.992 kWh, reflecting persistent I/O and memory overhead. In summary, Depthwise and Mixed Precision offered the best energy savings, while Group Convolution remained the most energy-intensive, with architectural overheads outweighing parameter reduction benefits.

Testing Phase: During inference, Standard Convolution proved to be the most energy-efficient configuration, with the lowest average energy consumption of 0.001427 kWh per test case. GPU, CPU, and RAM contributed 0.000777 kWh, 0.000303 kWh, and 0.000347 kWh, respectively, reflecting balanced hardware utilization and operational simplicity. In contrast, Depthwise Convolution recorded the highest energy consumption, averaging 0.001686 kWh per test case, with GPU energy at 0.001051 kWh and an average GPU power draw of 291.38 W. Fragmented channel-wise operations and frequent memory access offset expected energy efficiency gains. Group Convolution ranked in between, consuming 0.001479 kWh per test case. GPU energy reached 0.000808 kWh, with RAM and CPU contributing 0.000358 kWh and 0.000313 kWh, respectively. Despite parameter reduction, inefficient memory access and I/O operations led to elevated energy use, with GPU power averaging 212.58 W. In summary, Standard Convolution consistently delivered the lowest energy footprint during testing, while Depthwise and Group Convolutions experienced diminishing returns due to architectural overheads.

While this study offers detailed insights into energy-performance trade-offs, certain scope constraints introduce limitations that merit discussion. First, Mixed Precision optimization depends heavily on hardware support, particularly NVIDIA GPUs with Tensor Cores (e.g., Volta, Turing, Ampere), which limits the generalizability of energy savings to systems lacking such specialized accelerators. Performance gains may not translate to CPUs, older GPUs, or non-NVIDIA hardware, where FP16 operations could incur additional overhead. Second, the analysis is restricted to convolutional layer variations within a fixed U-Net architecture and does not include more advanced structural optimization methods such as neural architecture search (NAS), model pruning, or custom lightweight networks. While this design allowed controlled comparisons across convolution types and training strategies, it limited exploration into deeper architectural efficiencies. Third, although KiTS19 provided a controlled benchmark for CT-based kidney tumor segmentation, broader validation across multimodal, high-resolution datasets remains an important direction. We intentionally focused on KiTS19 because it is a well-annotated, real-world dataset with consistent training and testing splits, offering a stable foundation for measuring energy consumption across architectural and training configurations. While specific to abdominal CT, the observed trends, such as GPU energy dominance, Mixed Precision behavior, and inefficiencies in fragmented convolutions, are more reflective of underlying hardware behavior than dataset-specific characteristics. We therefore expect many of these patterns to generalize, though further validation is warranted. Fourth, all experiments were conducted offline and do not reflect clinical system-level conditions such as PACS integration, concurrent inference workflows, or patient-specific variability. Fifth, energy consumption was estimated using the CodeCarbon framework, which relies on software-based modeling rather than direct telemetry. While effective for benchmarking, these estimates should be interpreted as approximate values.

## 4. Conclusions

This study demonstrates the strong link between architectural design, optimization strategies, and energy efficiency in AI-based medical image segmentation. Depthwise Convolution with Mixed Precision emerged as the most energy-efficient configuration during training, while Standard Convolution proved most efficient during inference due to its computational simplicity. Mixed Precision consistently improved energy efficiency across all architectures. In contrast, Gradient Accumulation, despite its benefits for model stability, increased energy consumption due to longer training durations. Across all setups, the GPU remained the dominant source of energy usage, with RAM and CPU playing secondary roles. From a performance perspective, Standard Convolution delivered the most reliable segmentation accuracy, particularly for complex tumor regions. Depthwise and Group Convolutions, though more parameter-efficient, showed trade-offs in segmentation quality and energy stability. These findings underscore the importance of jointly optimizing training and inference workflows to build energy-conscious and scalable AI systems for medical image analysis. Future work should investigate techniques such as quantization to further reduce precision and energy costs without sacrificing accuracy. Additionally, efforts to optimize CUDA scheduling, mitigate GPU power spikes, and manage kernel execution more efficiently could further enhance energy stability. Extending this analysis to larger datasets, more complex architectures, and clinical deployment settings will provide deeper insights into the performance–sustainability trade-offs necessary for real-world AI adoption in healthcare.

These findings contribute meaningfully to the broader vision of sustainable AI in healthcare. As clinical AI systems scale, energy consumption should be considered a core design constraint, not an afterthought. This study demonstrates that even modest architectural and training-level changes can substantially impact energy demand, uncovering practical opportunities for reducing the environmental cost of deployment. Building on this, future work will extend our benchmarking framework to incorporate quantization-aware training, post-training quantization (e.g., INT8/FP8), and sparsity-aware strategies like structured pruning. To enhance measurement fidelity, we plan to integrate telemetry-based tools (e.g., NVML, RAPL) to capture real-time power draw and correlate it with compute patterns. A roadmap for sustainable clinical AI must include energy-aware model design, real-time monitoring, deployment aligned with clinical infrastructure, and green computing practices such as edge inference and workload scheduling. Frameworks like ours can support hospitals and regulatory bodies in quantifying the trade-offs between accuracy and energy use, laying the foundation for clinically reliable, low-carbon AI systems.

## Figures and Tables

**Table 1:** Experimental design illustrating diverse convolution methods, optimization approaches, and their implementation in training and testing phases.

Experiment	Model	Convolution Type	Optimization	Phase
1	U-Net	Standard Convolution	None	Training and Testing
2		Standard Convolution	Mixed Precision	Training
3		Standard Convolution	Gradient Accumulation	Training
4	U-Net	Depthwise Convolution	None	Training and Testing
5		Depthwise Convolution	Mixed Precision	Training
6		Depthwise Convolution	Gradient Accumulation	Training
7	U-Net	Group Convolution	None	Training and Testing
8		Group Convolution	Mixed Precision	Training
9		Group Convolution	Gradient Accumulation	Training

**Table 2:** Energy consumption for Standard, Depthwise, and Group Convolutions across configurations during training (NTO: No Training Optimization, MP: Mixed Precision, GA: Gradient Accumulation).

Metric	Standard Convolution			Depthwise Convolution			Group Convolution		
	NTO	MP	GA	NTO	MP	GA	NTO	MP	GA
Mean Duration (hr)	5.54	4.78	6.31	5.27	**4.44**	5.80	6.37	6.49	6.43
Mean Emissions (kg CO_2_)	0.5450	0.5336	0.6088	0.6028	**0.4811**	0.5534	0.6362	0.6376	0.6422
Mean GPU Power (W)	300.24	365.95	291.54	378.92	351.04	**287.38**	308.24	299.38	309.26
Mean CPU Energy (kWh)	0.4569	0.3942	0.5211	0.4345	**0.3659**	0.4783	0.5250	0.5359	0.5302
Mean GPU Energy (kWh)	1.6756	1.7546	1.8488	2.0053	**1.5598**	1.6706	1.9741	1.9579	1.9921
Mean RAM Energy (kWh)	0.5224	0.4507	0.5958	0.4968	**0.4183**	0.5469	0.6003	0.6127	0.6062
Mean Energy (kWh)	2.655	2.599	2.965	2.9367	**2.3441**	2.6959	3.0995	3.1066	3.1286
Total Energy (kWh)	5.209	5.099	5.802	5.7708	**4.6014**	5.2378	6.1030	6.0680	6.0900

Note: Values in bold represent the most efficient value for the specified metric.

**Table 3:** Per-epoch energy consumption for Standard, Depthwise, and Group Convolutions across configurations during training (NTO: No Training Optimization, MP: Mixed Precision, GA: Gradient Accumulation).

Metric	Standard Convolution			Depthwise Convolution			Group Convolution		
	NTO	MP	GA	NTO	MP	GA	NTO	MP	GA
Mean CPU Energy (kWh)	0.0179	0.0155	0.0204	0.0171	**0.0144**	0.0186	0.0207	0.0210	0.0206
Mean GPU Energy (kWh)	0.0658	0.0689	0.0724	0.0788	**0.0613**	0.0649	0.0777	0.0765	0.0776
Mean RAM Energy (kWh)	0.0205	0.0177	0.0233	0.0195	**0.0164**	0.0212	0.0237	0.0240	0.0236
Mean Total Energy (kWh)	0.1042	0.1020	0.1161	0.1154	**0.0920**	0.1048	0.1221	0.1214	0.1218
Mean Duration (sec)	782.3	674.5	889.6	745.54	**626.49**	810.49	903.0	914.2	899.0
Mean Emissions (kg CO_2_)	0.0214	0.0209	0.0238	0.0237	**0.0189**	0.0215	0.0251	0.0249	0.0250

Note: Values in bold indicate the most efficient value for the row.

**Table 4:** Energy consumption and performance metrics for Standard, Depthwise, and Group Convolutions during testing.

Metric	Standard Convolution	Depthwise Convolution	Group Convolution
Mean Energy Consumed (kWh)	0.001427	0.001686	0.001479
Mean GPU Power (w)	211.16	291.38	212.58
Mean CPU Energy (kWh)	0.000303	0.000243	0.000313
Mean RAM Energy (kWh)	0.000347	0.000392	0.000358
Mean Duration (90 cases)	13.24 min	12.95 min	13.67 min

**Table 5:** Segmentation performance metrics (Dice, Precision, Recall) for Standard, Depthwise, and Group Convolutions across configurations.

Metric	Standard Convolution			Depthwise Convolution			Group Convolution		
	NTO	MP	GA	NTO	MP	GA	NTO	MP	GA
Class 0 (Background)									
Dice	**0.9992**	0.9992	0.9992	0.9992	0.9991	0.9991	0.9991	0.9992	0.9991
Precision	**0.9997**	0.9996	0.9997	0.9996	0.9996	0.9997	0.9996	0.9996	0.9996
Recall	**0.9988**	0.9989	0.9986	0.9989	0.9985	0.9984	0.9986	0.9988	0.9986
Class 1 (Kidney)									
Dice	0.9020	**0.9055**	0.9052	0.9014	0.8988	0.9008	0.8937	0.8961	0.8945
Precision	0.8923	0.9005	**0.9010**	0.8985	0.8894	0.8967	0.8879	0.8918	0.8859
Recall	**0.9375**	0.9334	0.9310	0.9271	0.9332	0.9296	0.9252	0.9273	0.9286
Class 2 (Tumor)									
Dice	0.7189	**0.7332**	0.7246	0.6952	0.6728	0.6923	0.6663	0.6755	0.6712
Precision	**0.8802**	0.8720	0.8751	0.8672	0.8832	0.8715	0.8368	0.8446	0.8723
Recall	0.6932	**0.7115**	0.7006	0.6687	0.6303	0.6640	0.6439	0.6530	0.6332

Note: Values in bold indicate the most efficient value for the row.

## Data Availability

The dataset used in this study is publicly available as part of the Kidney Tumor Segmentation Challenge 2019 (KiTS19). It can be accessed through the Grand Challenge platform at https://kits19.grand-challenge.org/ (assessed on 1 May 2025). The dataset includes contrast-enhanced CT scans and corresponding ground truth annotations for kidney and tumor segmentation.

## Supplementary Materials

The following supporting information can be downloaded at: https://www.mdpi.com/article/10.3390/jimaging11060174/s1. Figure S1: Scatter plot showing (a) the linear relationship between the number of slices and energy consumed and (b) Inference duration and energy consumed; Figure S2: Cumulative energy breakdown during training for Standard Convolution, illustrating GPU dominance and supporting contributions from RAM and CPU; Figure S3: Per-slice energy consumption for Standard Convolution during inference; Figure S4: GPU power consumption across various training configurations; Figure S5: Training loss curves across various training configurations; Figure S6: Per-class segmentation metrics for Class 1 (Kidney) across all model configurations; Figure S7: Per-class segmentation metrics (Dice, Precision, Recall) for Class 2 (Tumor) across all model configurations. Section S1.1: CUDA Scheduling and Power Spikes during Training; Section S1.2: Training Loss Curves across U-Net Experiments; Section S1.3: Segmentation Behavior across Organs: Insights from Kidney and Tumor Regions.
