# Supplementary material for: A Study on Energy Consumption in AI-Driven Medical Image Segmentation
Source: J Imaging. 2025 May 26;11(6):174. doi: 10.3390/jimaging11060174 (PMC12194113; doi:10.3390/jimaging11060174)
Supplement: Supplementary file 1 [file jimaging-11-00174-s001.zip › jimaging-3621439-supplementary.pdf]

A Study on Energy Consumption in AI-Driven Medical Image Segmentation

R. Prajwal [1], S. J. Pawan [1,*], Shahin Nazarian [2], Nicholas Heller [3], Christopher J. Weight [3,4], Vinay Duddalwar [1,5,6,7] and C.-C. Jay Kuo [2]

[1] Radiomics Lab, University of Southern California, Los Angeles, CA 90033, USA; prajwalr0411@gmail.com (R.P.); vinay.duddalwar@med.usc.edu (V.D.)
[2] Ming Hsieh Department of Electrical and Computer Engineering, University of Southern California, Los Angeles, CA 90089, USA; shahin.nazarian@usc.edu (S.N.); jckuo@usc.edu (C.-C.J.K.)
[3] Glickman Urological Institute, Cleveland Clinic, Cleaveland, OH 44125, USA; helle246@umn.edu (N.H.); weightc@ccf.org (C.J.W.)
[4] Cleveland Clinic Lerner, School of Medicine, College of Medicine of Case, Western Reserve University, Cleveland, OH 44106, USA
[5] Alfred E Mann Department of Biomedical Engineering, University of Southern California, Los Angeles, CA 90089, USA
[6] Institute of Urology, University of Southern California, Los Angeles, CA 90033, USA
[7] Department of Radiology, Los Angeles General Medical Center, Los Angeles, CA 90033, USA
* Correspondence: pawan.jogi@med.usc.edu

Supplementary Material

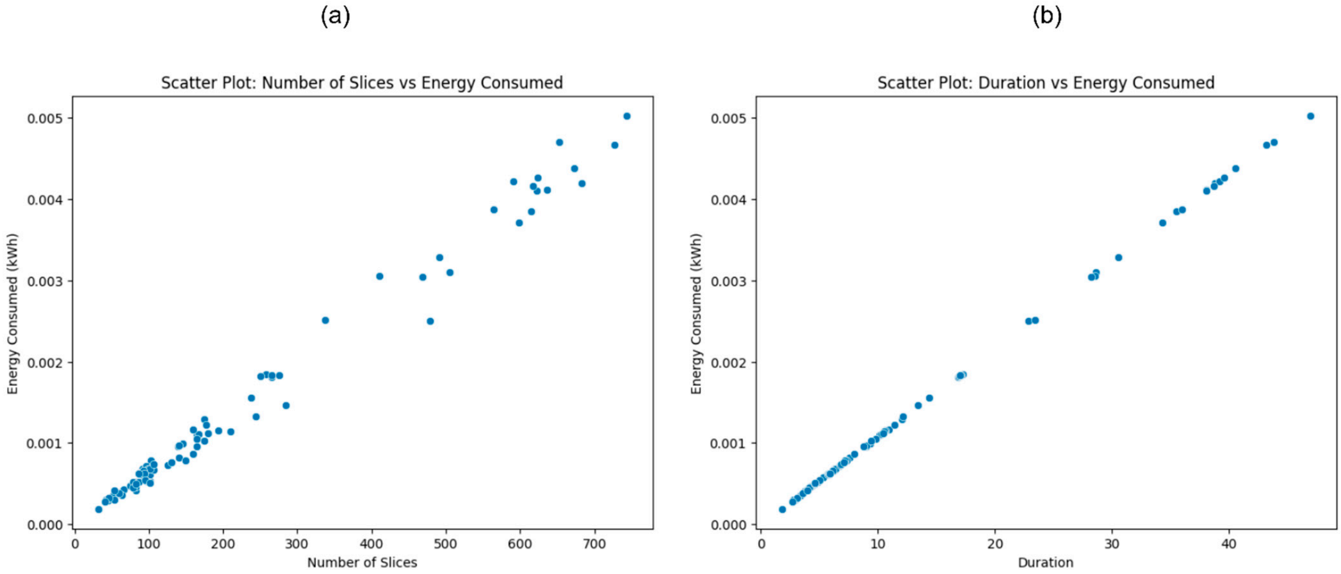

Figure S1: Scatter plot showing a) the linear relationship between the number of slices and energy consumed and b) Inference duration and energy consumed.

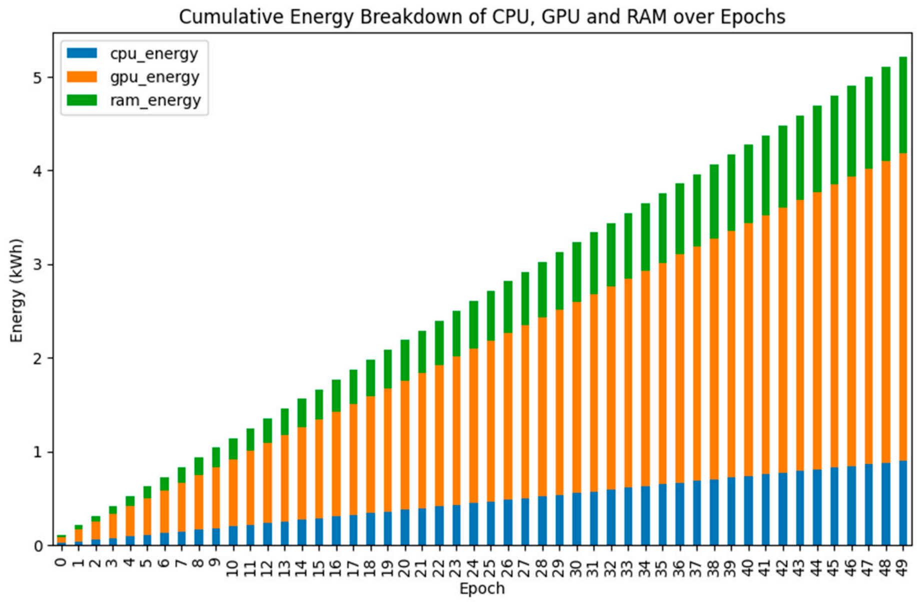

Figure S2: Cumulative energy breakdown during training for Standard Convolution, illustrating GPU dominance and supporting contributions from RAM and CPU.

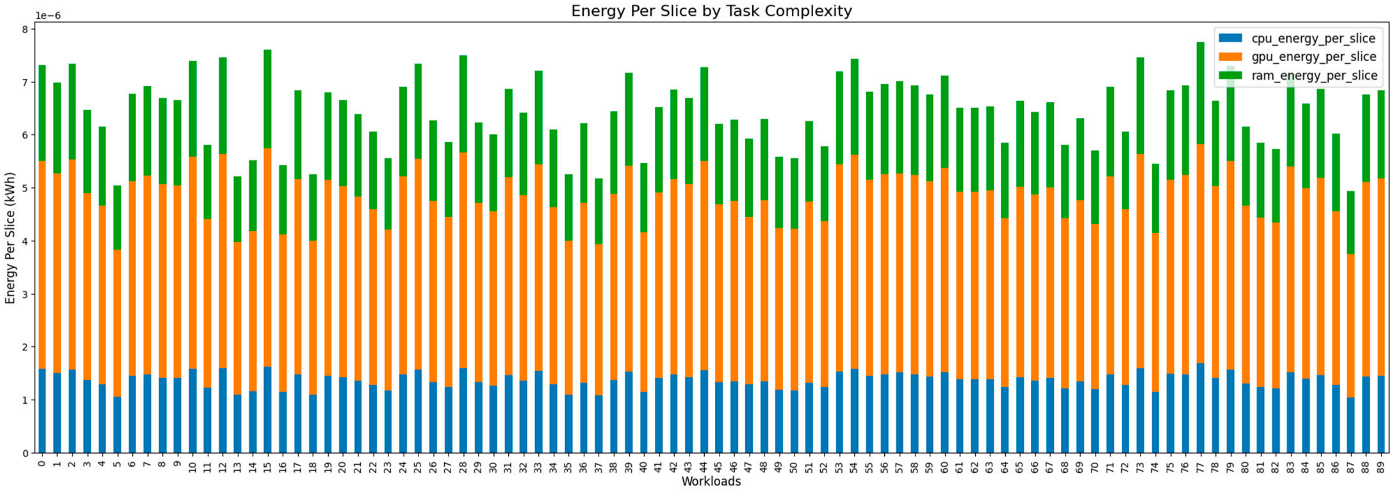

Figure S3: Per-slice energy consumption for Standard Convolution during inference.

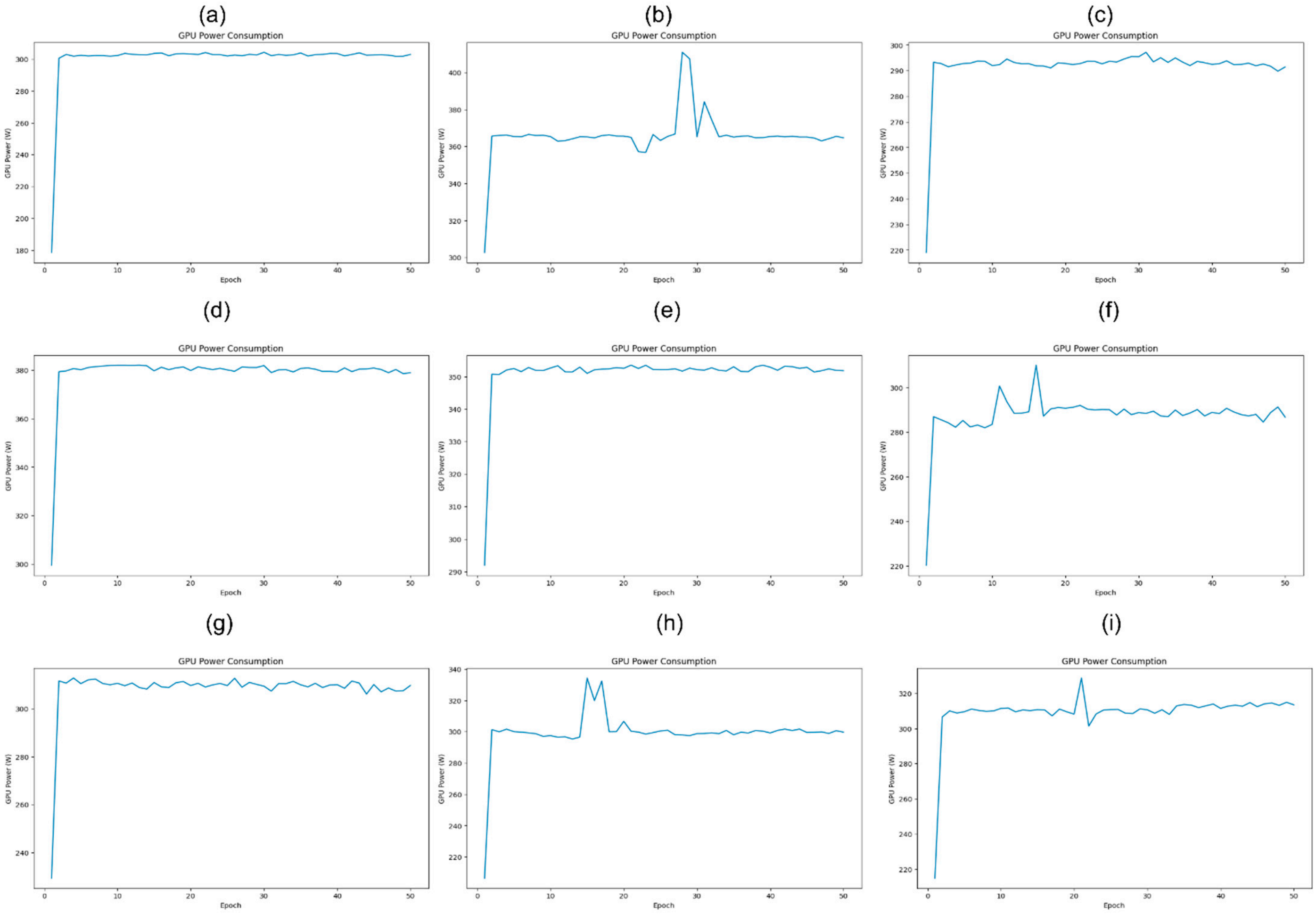

Figure S4: GPU power consumption across various training configurations. (a) Standard Convolution during training without optimization, (b) Standard Convolution during training with Mixed Precision, (c) Standard Convolution during training with Gradient Accumulation, (d) Depthwise separable convolution during training without Optimization, (e) Depthwise separable convolution during training with Mixed Precision, (f) Depthwise separable convolution during training with Gradient Accumulation, (g) Group Convolution during training without Optimization (h) Group Convolution during training with Mixed Precision (i) Group Convolution during training with Gradient Accumulation.

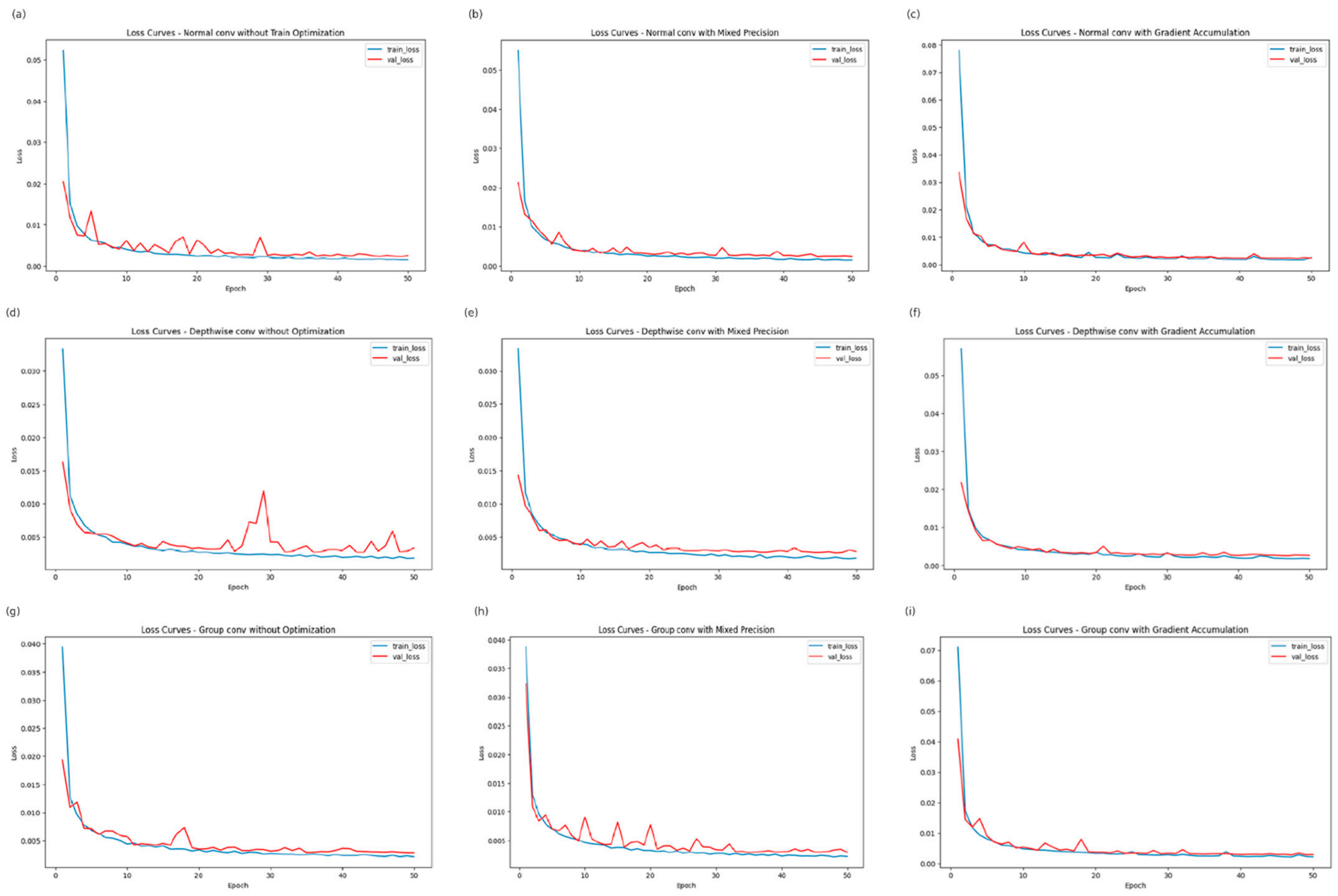

Figure S5: Training loss curves across various training configurations. (a) Standard (Normal) Convolution during training without optimization, (b) Standard (Normal) Convolution during training with Mixed Precision, (c) Standard (Normal) Convolution during training with Gradient Accumulation, (d) Depthwise separable convolution during training without Optimization, (e) Depthwise separable convolution during training with Mixed Precision, (f) Depthwise separable convolution during training with Gradient Accumulation, (g) Group Convolution during training without Optimization (h) Group Convolution during training with Mixed Precision (i) Group Convolution during training with Gradient Accumulation.

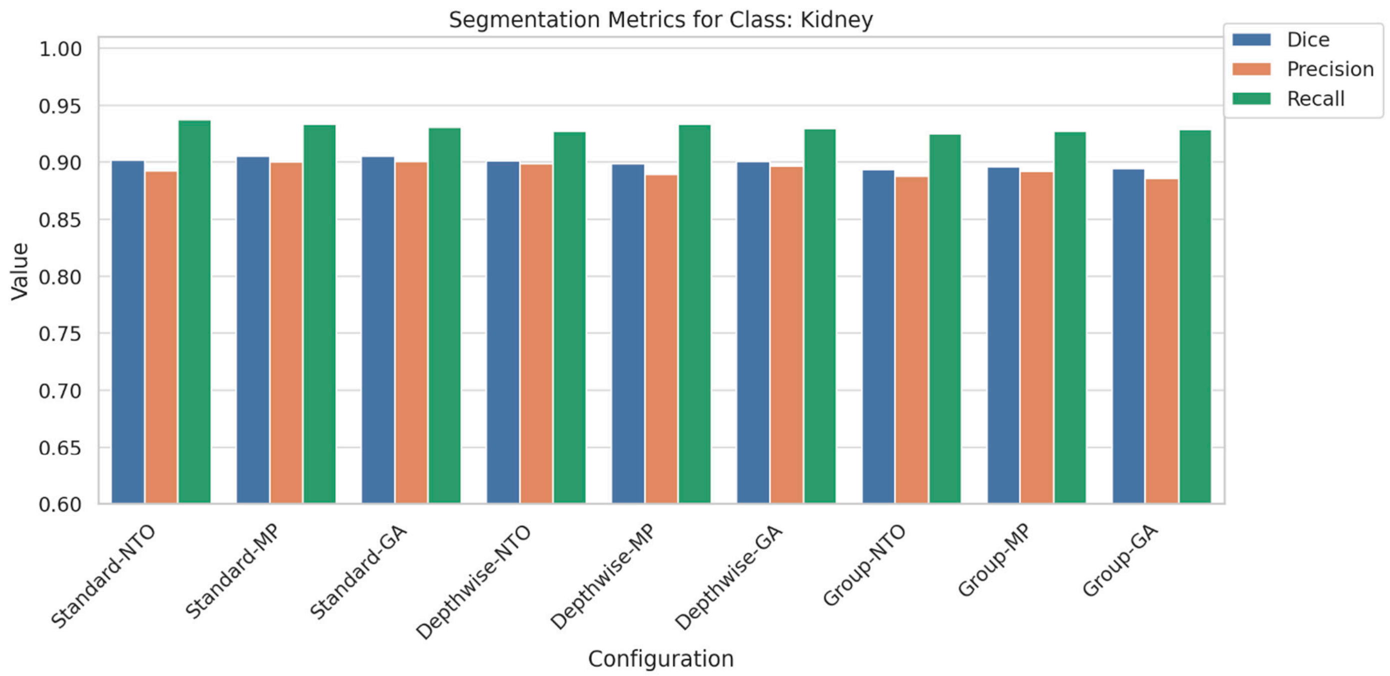

Figure S6: Per-class segmentation metrics (Dice, Precision, Recall) for Class 1 (Kidney) across all model configurations.

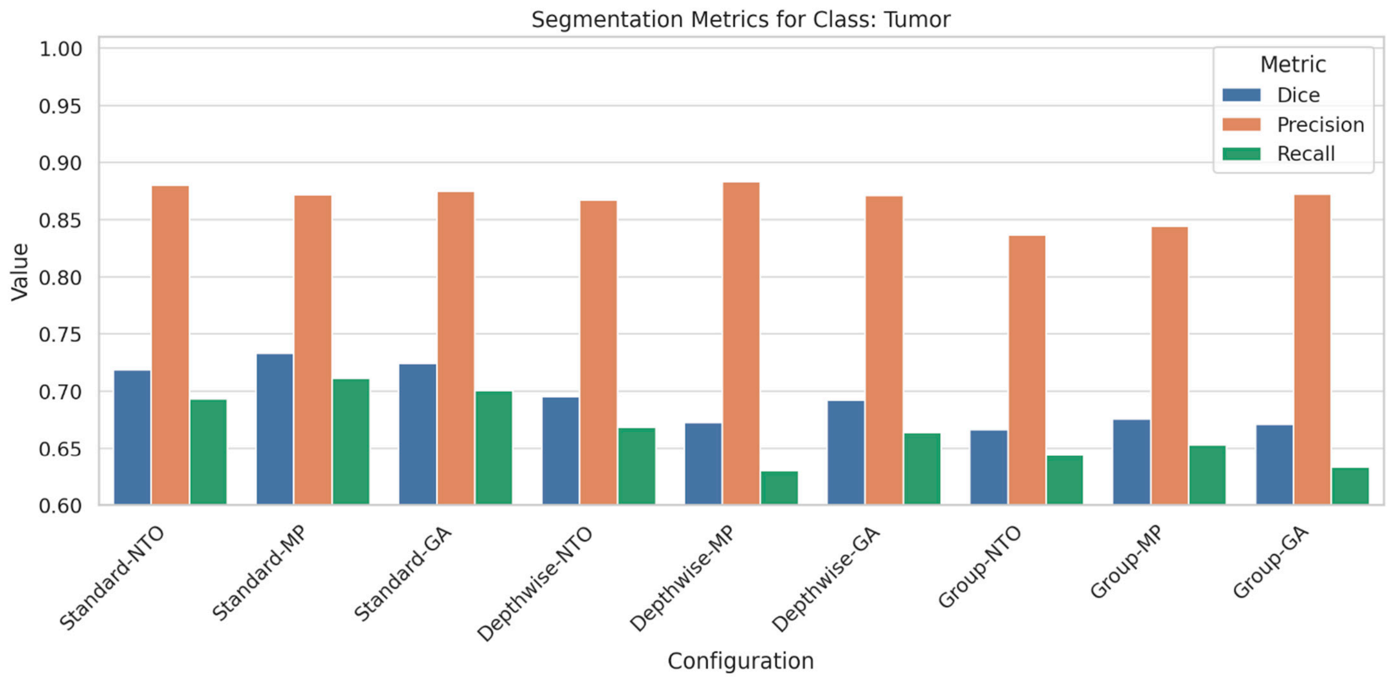

Figure S7: Per-class segmentation metrics (Dice, Precision, Recall) for Class 2 (Tumor) across all model configurations.

1.1 CUDA Scheduling and Power Spikes during Training

The analysis of GPU power consumption revealed notable power spikes across configurations, particularly in Mixed Precision setups and Gradient Accumulation, as illustrated in Figure S4 (b), Figure S4 (f), Figure S4 (h), and Figure S4 (i). These power fluctuations can be attributed to the interplay between CUDA scheduling, kernel execution patterns, and synchronization overhead. In Mixed Precision training (Figure S4 (b) and Figure S4 (h)), spikes exceeding 400 W were observed, particularly during early epochs and computationally intensive phases. These spikes are caused by the dynamic switching between FP16 and FP32 kernels, which introduces scheduling delays and queuing inefficiencies. FP32 kernels, being more computationally intensive, require additional GPU resources, leading to temporary surges in power demand. Furthermore, the frequent kernel launches in depthwise and Group Convolutions exacerbate scheduling challenges. In Depthwise Convolutions, each input channel is processed independently, resulting in numerous lightweight kernels, while Group Convolutions divide input channels into smaller groups, requiring separate kernels for each group. These fragmented operations introduce significant overhead, as the GPU scheduler must repeatedly allocate resources, manage memory, and synchronize threads for every kernel. The interchanging of FP16 and FP32 kernels in Mixed Precision setup further complicates scheduling, disrupting CUDA's execution flow and causing delays and queuing inefficiencies. Additionally, irregular memory access patterns amplify these challenges, creating bottlenecks and temporary power surges as the GPU attempts to maintain computational stability. For Gradient Accumulation setups (Figure S4 (f) and Figure S4 (i)), power spikes can be attributed to synchronization challenges associated with the accumulation process. These challenges arise from the need to delay weight updates until gradients from multiple mini-batches are aggregated, introducing dependencies and potential bottlenecks at synchronization points. During these epochs, slight delays or backlogs in the data pipeline or gradient synchronization caused temporary overloads as the GPU caught up with pending computations. Gradient accumulation introduces additional overhead by aggregating gradients over multiple mini-batches before weight updates, which can increase the computational load momentarily. These isolated spikes disrupt the stable power consumption profile, as seen in Figure S4 (i) where power consistently hovered around 300 W, except during synchronization-induced spikes. Overall, the spikes observed in Mixed Precision and Gradient Accumulation setups underscore the challenges posed by CUDA scheduling in managing dynamic kernel execution and synchronization demands. The interplay between FP16 and FP32 kernels, frequent kernel launches in fragmented architectures, and backlogged gradient updates contribute to these fluctuations. Addressing these inefficiencies through improved CUDA stream management and memory handling strategies could help stabilize GPU power usage and enhance computational efficiency across training configurations.

1.2 Training Loss Curves across U-Net Experiments

For the Standard Convolution U-Net, all configurations demonstrated effective convergence, with nuanced differences in optimization behavior. In the unoptimized setup (Figure S5a), both training and validation losses dropped rapidly within the first 10 epochs and gradually plateaued. A consistent, small gap between the two curves suggested mild overfitting, with occasional validation spikes beyond epoch 20 likely reflecting variability in slice complexity. When trained with Mixed Precision (Figure S5b), the model exhibited faster stabilization, converging within approximately 15 epochs, and maintained slightly lower loss values throughout. Importantly, Mixed Precision did not introduce any instability, confirming its compatibility with the standard architecture. The Gradient Accumulation variant (Figure 5c) yielded the smoothest loss trajectories among all, with a slower, more controlled descent in loss values. This stability reflects the benefits of simulating a larger batch size, which reduced noise in gradient updates and improved convergence consistency. Overall, the standard U-Net maintained robust training dynamics under all three strategies, with Gradient Accumulation offering the most regularized optimization profile. For the Depthwise Separable Convolution U-Net, training dynamics varied noticeably across optimization strategies. In the unoptimized configuration (Figure S5d), early convergence was achieved; however, the validation loss exhibited significant fluctuations between epochs 25–40. This instability likely stems from the fragmented per-channel processing and the reduced representational capacity intrinsic to depthwise convolutions. With Mixed Precision (Figure S5e), the convergence behavior improved substantially, loss curves were smoother, and the model stabilized faster without any indication of numerical instability from FP16 operations. After epoch 15, validation loss closely tracked the training loss, suggesting better generalization. The Gradient Accumulation variant (Figure S5f) delivered the most stable and lowest loss trajectories among the depthwise configurations. The close alignment of training and validation losses reflects reduced gradient noise and indicates that accumulation effectively compensates for the sparse computation overhead of depthwise layers, resulting in improved optimization stability. For the Group Convolution U-Net, all configurations demonstrated consistent convergence with distinct stability characteristics. In the unoptimized setup (Figure S5g), the model showed stable loss descent with minimal oscillations, and validation closely followed training loss, albeit with slightly slower convergence compared to the standard convolution baseline, likely due to the structured sparsity introduced by grouped filters. With Mixed Precision (Figure S5h), both training and validation losses declined rapidly and stabilized effectively. However, intermittent spikes in validation loss between epochs 10–30 were noted, potentially stemming from memory alignment inefficiencies during grouped operations under low-precision computation. Despite this, convergence remained robust overall. The Gradient Accumulation variant (Figure S5i) achieved the most stable optimization among all group convolution configurations, characterized by smooth, closely aligned loss curves and reduced variance. While convergence was more gradual, the setup benefited from the regularizing effect of larger effective batch sizes, enhancing training consistency without compromising the advantages of grouped architectural design.

1.3 Segmentation Behavior across Organs: Insights from Kidney and Tumor Regions

As shown in Figure S6, the Standard Convolution U-Net variants consistently demonstrate superior segmentation performance for the Kidney class across all three evaluation metrics, Dice, Precision, and Recall. Within this family, configurations incorporating Mixed Precision (MP) and Gradient Accumulation (GA) yield marginal but consistent improvements over the No Train Optimization (NTO) baseline. These optimizations appear to enhance the model's capacity to delineate organ boundaries more precisely, as reflected in the elevated Dice and Precision scores. In contrast, models employing Depthwise Separable and Group Convolution show a slight decline in Dice and Precision, suggesting a trade-off in representational expressiveness introduced by parameter-reduction strategies. Despite these variations, Recall values remain high and relatively stable across all configurations, indicating that the models, regardless of architectural modifications, retain a strong ability to correctly identify kidney regions. This robustness in sensitivity suggests that architectural sparsity affects boundary precision more than it impacts detection capability. As shown in Figure S7, tumor segmentation poses the greatest challenge across all model configurations, largely due to the small size and irregular shape of tumor regions. Standard Convolution U-Nets, especially when combined with Mixed Precision (MP) and Gradient Accumulation (GA), consistently yield the highest Dice and Recall scores, demonstrating superior ability to detect and delineate sparse tumor structures. On the other hand, Depthwise and Group Convolution variants show a noticeable drop in Recall, reflecting difficulty in capturing the full extent of tumor boundaries. Interestingly, Precision remains relatively high across all models, indicating that most predicted tumor pixels are correct, but a significant portion of actual tumor pixels are being missed. This discrepancy between Precision and Recall suggests that many models lean toward conservative segmentation, minimizing false positives at the cost of increased false negatives. These findings highlight the critical need to optimize both sensitivity and specificity when designing models for tumor segmentation in clinical applications.
